# Paroxetine overdose during pregnancy

**DOI:** 10.1080/20961790.2021.1938802

**Published:** 2021-09-28

**Authors:** Selin Acar, Hilal Erol, Elif Keskin Arslan, Nusret Uysal, Barış Karadaş, Tijen Kaya Temiz, Yusuf Cem Kaplan

**Affiliations:** a Faculty of Medicine, Department of Pharmacology, Izmir Katip Celebi University, Izmir, Turkey; b Terafar (Izmir Katip Celebi University Teratology Information, Training and Research Center), Izmir, Turkey

**Keywords:** Forensic sciences, forensic toxicology, paroxetine, overdose, teratogenicity, case report

## Abstract

Paroxetine is a selective serotonin reuptake inhibitor (SSRI) used in the treatment of depression and anxiety disorders. In some epidemiological studies, slightly increased risks of major malformations and cardiac malformations have been reported following paroxetine exposure in the first trimester of pregnancy. However, such findings have been inconsistent. There is only one report of any overdose of an SSRI during pregnancy, and that involved escitalopram. The aim of this case report was to describe the impact of a paroxetine overdose in the first trimester of pregnancy on the health of the foetus. A 21-year-old mother of one child who was pregnant with a second child was prescribed 20 mg/day paroxetine hydrochloride for the treatment of anxiety/depression. The patient ingested 15 or 16 20-mg tablets of paroxetine hydrochloride (300–320 mg) during the 5th week of pregnancy as a suicide attempt. Within 15 min of ingestion, she was admitted to hospital and treated for intoxication. No evidence of maternal SSRI intoxication was observed after treatment. The patient consulted our teratology information service for further risk assessment regarding possible major congenital malformations following the paroxetine overdose. We were unable to find previous reports of paroxetine overdose during pregnancy in the literature. The timely administration of the overdose treatment and the lack of maternal intoxication symptoms were considered positive for the foetal well-being, and the patient was referred for perinatology and psychiatry follow-ups. A healthy, 3 500-g male infant was born at 38 weeks’ gestation, and his development at the age of 2 years was normal. This is the first reported case of paroxetine overdose during pregnancy. Comprehensive studies are needed to evaluate pregnancy outcomes after SSRI overdose.

**Key Points**
There are no reported data on paroxetine overdose during pregnancy.
The aim of this case report was to describe the impact of a maternal paroxetine overdose in the first trimester of pregnancy on the health of the foetus. No evidence of maternal SSRI intoxication was observed.
No congenital malformations or developmental disorders were observed in the child at 2 years of age.
Comprehensive studies are needed to evaluate pregnancy outcomes following SSRI overdose.

## Introduction

Paroxetine is a selective serotonin reuptake inhibitor (SSRI) used in the treatment of depression and anxi­ety disorders. In a 2016 meta-analysis, it was reported that there were increased risks of any major congenital malformation (pooled odds ratio (OR): 1.23; 95% confidence interval (CI): 1.10–1.38; *n* = 15 studies) and major cardiac malformations (pooled OR: 1.28; 95%CI: 1.11–1.47; *n* = 18 studies) following paroxetine exposure in the first trimester of pregnancy [1]. However, the risk estimates differed depending on the characteristics of the comparison group. For example, the risk of cardiac malformations was not significantly higher than a disease-matched control group including women who were diagnosed with depression and/or anxiety but not exposed to any antidepressant. Further ana­lysis revealed that there were no significant increases in the rates of major congenital or cardiac malformations when the exposed group was compared with disease-matched controls with or without antidepressant exposure (other than paroxetine), which suggested a possible bias by indication (confounded by underlying disease). Several studies have exa­mined the impact of SSRI dose. Increased risks of major congenital malformations (adjusted OR: 2.23; 95%CI: 1.19–4.17) and major cardiac malformations (adjusted OR: 3.07; 95%CI: 1.00–9.42) were found following exposure to >25 mg/day paroxetine during the first trimester of pregnancy [2]. Exposure to a high dose of SSRIs was also associated with lower gestational age (*P* = 0.009) and a higher rate of prematurity (OR: 5.07; 95%CI: 1.34–19.23) [3]. To our knowledge, there is only one case report of an SSRI overdose during pregnancy [4, 5]. In that case, a 36-year-old pregnant woman ingested an overdose of escitalopram (280 mg) during the 31st week of amenorrhoea. The patient was given activated charcoal-carbomix 5 h after the intoxication. Because the patient presented a risk of premature delivery, tocolytic treatment was also given. The child was born spontaneously at 37 weeks and 4 days’ gestation, with no indication of respiratory distress, renal failure, vomiting or convulsions. The infant exhibited extreme agitation, constant irritability and significant nervousness that dissipated during a 17-day stay in hospital; consequently, the mother and the infant were discharged [4]. To date, there are no published data on paroxetine overdose during pregnancy. The aim of this case report was to describe the impact of a paroxetine overdose in the first trimester of pregnancy on the health of the foetus.

## Case

A 21-year-old mother of one child who was pregnant with her second child was prescribed 20 mg/day paroxetine hydrochloride for the treatment of anxiety/depression. The patient ingested 15 or 16 20-mg tablets of paroxetine hydrochloride (300–320 mg) during the 5th week of pregnancy as a suicide attempt but was admitted to hospital within 15 min. A nasogastric tube was inserted, gastric lavage was applied and activated charcoal was given. The patient was monitored closely for 30 min and then discharged. No symptoms indicative of perfusion disorder or somnolence resulting from central nervous system depression, such as hypotension or syncope, occurred within the following 24 h. The patient was referred to Terafar-Izmir Katip Celebi University Teratology Information, Training and Research Center for further risk assessment of possible major congenital malformations. At her initial consultation she was at 10 weeks and 3 days’ gestation. Data and literature searches performed on the Reprotox® and PubMed databases, as well as the third edition of *Drugs During Pregnancy and Lactation: Treatment Options and Risk Assessment* [5], failed to retrieve any data on paroxetine overdose during pregnancy. The appropriateness of the patient’s intoxication treatment and lack of symptoms were considered to be positive signs for the continued healthy development of the foetus. Psychiatric follow-up and perinatological assessment (detailed ultrasonography and foetal ecocardiography) were recommended. In the postnatal follow-up *via* telephone call, the mother reported that a healthy 3 500-g male infant was born at 38 weeks’ gestation *via* caesarean section, and that the child’s development at the age of 2 years was normal.

## Discussion

To the best of our knowledge, this is the first report of a case of paroxetine overdose during pregnancy. There is one case report describing an SSRI overdose in pregnancy, and that involved escitalopram in the third trimester [4, 5], for which treatment for intoxication was administered 5 h later [4]. Bérard et al. [2] reported increased risks of major congenital and major cardiac malformations following maternal ingestion of >25 mg/day of paroxetine. However, it is not known whether any of the patients in that study used short-term. In another study, Roca et al. [3] reported that the exposure to relatively higher dose of an SSRI was associated with lower gestational age and higher rates of prematurity. In our case, there was no evidence of SSRI intoxication in a woman exposed to a paroxetine overdose in the first trimester of pregnancy, nor were there any congenital malformations or deve­lopmental disorders in the infant up to 2 years later. Given the widespread use of the SSRIs, it is noteworthy that this is the first reported case of pa­roxetine overdose during pregnancy. Comprehensive studies are needed to evaluate pregnancy outcomes after SSRI overdose.
